# Background parenchymal enhancement of the contralateral breast on preoperative contrast-enhanced breast MRI as a potential predictive factor for disease-free survival in triple-negative breast cancer patients

**DOI:** 10.3389/fonc.2025.1700320

**Published:** 2025-10-21

**Authors:** Xiao-Ting Li, Xing Wang, Hai-Tao Zhu, Nan Sun, Hai-Bin Zhu, Liang You, Xiao-Lei Gu, Yao Luo, Zhao-Qing Fan, Ying-Shi Sun

**Affiliations:** ^1^ Key Laboratory of Carcinogenesis and Translational Research (Ministry of Education/Beijing), Department of Radiology, Peking University Cancer Hospital & Institute, Beijing, China; ^2^ Key Laboratory of Carcinogenesis and Translational Research (Ministry of Education/Beijing), Breast Center, Peking University Cancer Hospital & Institute, Beijing, China

**Keywords:** breast neoplasms, magnetic resonance imaging, background parenchymal enhancement, disease-free survival, neoadjuvant chemotherapy

## Abstract

**Background:**

Background parenchymal enhancement (BPE) observed on dynamic contrast-enhanced (DCE) MRI of the contralateral breast is considered to be associated with survival outcomes. However, the prognostic significance of BPE in triple-negative breast cancer (TNBC) is unclear.

**Methods:**

Between March 2017 and June 2019, 76 TNBC patients undergoing neoadjuvant therapy and subsequent surgery were included in the study. All patients underwent DCE MRI before and after neoadjuvant therapy. Radiologists graded BPE as minimum, mild, moderate, and marked. The BPE level was analyzed according to clinicopathological characteristics and MRI findings. Survival analysis was conducted for clinicopathological characteristics and MRI findings according to disease-free survival (DFS).

**Results:**

The mean age was 51.29 ± 9.53 years; 46 (60.5%) patients achieved pathological complete response (pCR), and 13 (17.1%) patients developed recurrence, with a median follow-up of 80 months (interquartile range: 64, 90). Dichotomous BPE (minimal/mild vs. moderate/marked) on post-NAC MRI was statistically associated with post-NAC ADC and menopausal status. Patients with BPE changing from high to low level demonstrated statistically lower recurrence rate than patients with BPE changing from low to high (*P* = 0.022). BPE on post-NAC MRI was in the final multivariate Cox model for DFS (HR = 6.57, minimal/mild: HR = 1), along with multifocality on post-NAC MRI (HR = 3.65, no multifocality: HR = 1) and pCR (HR = 7.27, pCR: HR = 1).

**Conclusion:**

Contralateral BPE and its change after neoadjuvant chemotherapy may reflect the recurrence risk in triple-negative breast cancer patients.

## Introduction

Background parenchyma enhancement (BPE) refers to the enhancement manifestation of the normal breast tissue in breast dynamic contrast-enhanced (DCE) magnetic resonance imaging (MRI) examination (1). BPE is usually evaluated according to a four-point scale of minimum, mild, moderate, and marked (1). High BPE can reflect an increase in vascular permeability, promote angiogenesis in the tumor microenvironment, and accelerate tumor growth and metastasis (2). Previous studies have proved that increased BPE can serve as a marker for developing breast cancer (3, 4) and may indicate a higher risk for the recurrence of breast cancer (5–7).

BPE is affected by estrogen levels, the menstrual cycle, menopausal status, and hormone replacement therapy (8, 9). Therefore, high BPE may indicate a poor response to endocrine therapy, potentially linked to the hormone-dependent proliferation pathway. This suggests that the prognostic value of BPE may be more significant in hormone receptor-positive breast cancer. In triple-negative breast cancer (TNBC), studies provided inconsistent evidence, whether high BPE was associated with a worse prognosis (10, 11), which may be related to the heterogeneity of TNBC and its relatively low dependence on the hormonal microenvironment. In addition, there are relatively few studies focusing on the dynamic changes of BPE after treatment according to survival outcomes in TNBC.

In summary, although BPE is a potential prognostic factor for breast cancer, its characteristics in TNBC and whether it may serve as a non-invasive imaging biomarker for risk stratification remain unknown at present. Therefore, this study proposed a retrospective analysis to evaluate whether BPE of the contralateral breast on breast MRI before and after neoadjuvant chemotherapy (NAC) and its dynamic changes are associated with primary breast cancer features and disease-free survival outcomes in TNBC patients.

## Materials and methods

### Patients

This study was approved by our institutional review board, and the patient informed consent was waived due to the retrospective design. Data from patients with operable TNBC confirmed by histology were retrospectively collected between March 2017 and June 2019 from the database of Peking University Cancer Hospital. The inclusion criteria include the following: age ≥18 years, received NAC before surgery, and received DCE MRI before and after NAC. The exclusion criteria were as follows: bilateral breast cancer, quality of MRI not meeting the requirements for evaluation, incomplete clinicopathological data, and lost to follow-up after surgery.

All tumors were evaluated by immunohistochemistry (IHC) for estrogen (ER) and progesterone (PR) receptors as well as for Her-2/neu by IHC and/or fluorescence *in situ* hybridization (FISH). TNBC was defined by a finding of ER and PR <1% and Her-2 (0, 1+, or 2+). If Her-2 was expressed at 2+, then the follow-up FISH should be negative. Clinicopathologic data included age, NAC regimen, pathological type, original state of axillary lymph node (LN), and pathological complete response (pCR) status. Axillary LN status was defined by needle biopsy as pN+ and pN− before NAC. Pathological complete response was defined as no invasive residual cancer cells in the breast (ypTis/0) from the total samples. LNpCR was defined as no residual cancer cells in the axillary LNs (ypN0) only from the pre-NAC pN+ samples.

### MR examination

MR examinations were carried out within 2 weeks before NAC and 2 weeks before surgery for each patient. All breast MRI examinations were performed using a 1.5T system (GE Optima MR360; GE Healthcare,Tianjin,China; GE Healthcare) equipped with an 8-channel breast coil (GE Healthcare, Tianjin,China; GE Healthcare) with patients in the prone position.

Firstly, axial T2-weighted, fat-suppressed, short inversion time inversion recovery sequences were performed (TR = 5,000–5,800 ms, TE = 63.49 ms, TI = 160 ms, slice thickness = 4 mm, no interlayer gap, matrix size = 256 × 256, field of view = 28~36 cm, NEX = 2). Secondly, axial DWI examinations were performed using a diffusion-weighted echo planar imaging sequence with *b*-values of 0 and 1,000 s/mm (TR = 8,000 ms, TE = 79.7 ms, field of view = 32 × 18 cm, matrix = 150 × 80, slice thickness = 4.0 mm). Diffusion gradients were applied in three orthogonal directions.

Then, a dynamic enhanced axial three-dimensional vibrant SPGR sequence (TR = 6.4 ms, TE = 3.0 ms, TI = 7.0 ms, flip angle = 15°, slice thickness = 2.2 mm, with 50% overlap, matrix size = 320 × 320, field of vision = 28–36 cm, sequential K space filling, scan time per acquisition = 60 s). The sequence was repeated six times, with the first phase acquired before contrast enhancement and the other five phases acquired after contrast enhancement. The contrast agent (Gd-DTPA) was injected into the anterior elbow vein by a power syringe at a speed of 2.0 mL/s based on the patient’s weight (0.2 mmol/kg) and flushed with 20 mL of saline. The injection of the contrast agent and the second phase started at the same time.

### Image evaluation

All MRI images were retrospectively obtained and assessed by a radiologist (H.B.Z.), who was blinded to the clinicopathologic and follow-up data. Two experienced radiologists (H.B.Z. and X.L.G.) independently conducted the BPE evaluation. The contralateral normal breast was used for image analysis. BPE was qualitatively assessed based on the intensity and volume of enhancement of normal fibroglandular tissues, using four categories defined by the Breast Imaging Reporting and Data System (BI-RADS) atlas: minimal, mild, moderate, or marked (1). In this study, BPE was assessed at the early arterial phase (first enhancement phase, in which the central K space time was approximately 30 s after contrast agent administration) in accordance with Breast Imaging Reporting and Data System (BI-RADS) Magnetic Resonance Imaging (MRI) lexicon (12). A third senior radiologist (Y.S.S.) was consulted to resolve discrepancies. DCE curve type (outflow/plateau/inflow), apparent diffusion coefficient (ADC value calculated from diffusion-weighted imaging with *b*-values of 0 and 1,000 s/mm), multifocality (yes/no), morphology (mass/non-mass), and maximum diameter (cm) were evaluated by the third radiologist.

### Treatment and follow-up data

Two NAC regimens were used. The dose-dense (ddEC-wP) involved 4 cycles of epirubicin and cyclophosphamide every 2 weeks and then 12 weeks of paclitaxel weekly, with prophylactic pegfilgrastim or rhG-CSF. The conventional (EC-wP) had the same drugs/doses but at 3-week intervals for 4 cycles, with no prophylaxis, and then 12 weeks of paclitaxel weekly.

The surgical method depended on the patient’s will and the medical evaluation after NAC. Axillary LN dissection (ALND) was conducted for those with positive LN. Radiotherapy was conducted for patients undergoing breast-conserving surgery (BCT) or positive LN after NAC.

The primary endpoint was disease-free survival (DFS). DFS was calculated from the data of neoadjuvant treatment to the earliest occurrence of local recurrence, distant relapse, or death without prior relapse. In cases where the disease spread to the contralateral breast simultaneously with local and/or other distant site recurrences, it was regarded as a relapse. However, if the disease emerged solely in the contralateral breast without any local or distant recurrence, it was classified as a second primary cancer and not counted as a DFS failure. The follow-up time was censored at the last follow-up date for patients without follow-up events. All patients were followed according to a uniform institutional protocol. During the first 2 years postoperatively, follow-up was conducted every 3 months. From year 2 to year 5, follow-up was performed every 6 months. Beyond 5 years, annual follow-up was carried out. At each visit, surveillance included physical examination, breast/chest wall/axillary ultrasonography, imaging of the chest and abdomen (via CT or ultrasonography), and serum tumor marker assessment.

### Statistical analysis

Normally distributed continuous variables were represented by means and standard deviation, while non-normally distributed continuous variables were represented by median and interquartile range (IQR). Categorical variables were represented by numbers. The independent sample *t*-test or Mann–Whitney test was used for comparisons of continuous variables between groups. Comparisons of categorical variables were conducted using the chi-square test or Fisher’s exact test. Cohen’s weighted kappa index (*κ*) was used to evaluate interobserver agreement, with 0.0–0.20, 0.21–0.40, 0.41–0.60, 0.61–0.80, and 0.81–1.00 indicating poor, fair, moderate, substantial, and excellent agreement, respectively (13). Univariate and multivariate Cox regression analyses were conducted to detect the prognostic effect of patient characteristics and MRI assessments according to DFS. Before the multivariate Cox regression was conducted, multicollinearity was detected. If the variance inflation factor (VIF) ≥10, strong multicollinearity was considered to exist, and then the variable with a lower *P*-value in the univariate analysis was retained for further multivariate analysis. Hazard ratios (HRs) with 95% confidence intervals were calculated. Kaplan–Meier curves with log-rank estimates were applied for the BPE groups. Two-sided *P*-values less than 0.05 were considered statistically significant. All statistical analysis was conducted using R4.4.2(R Core Team,Vienna, Austria).

## Results

### Patient characteristics and disease recurrences

**Figure 1:**
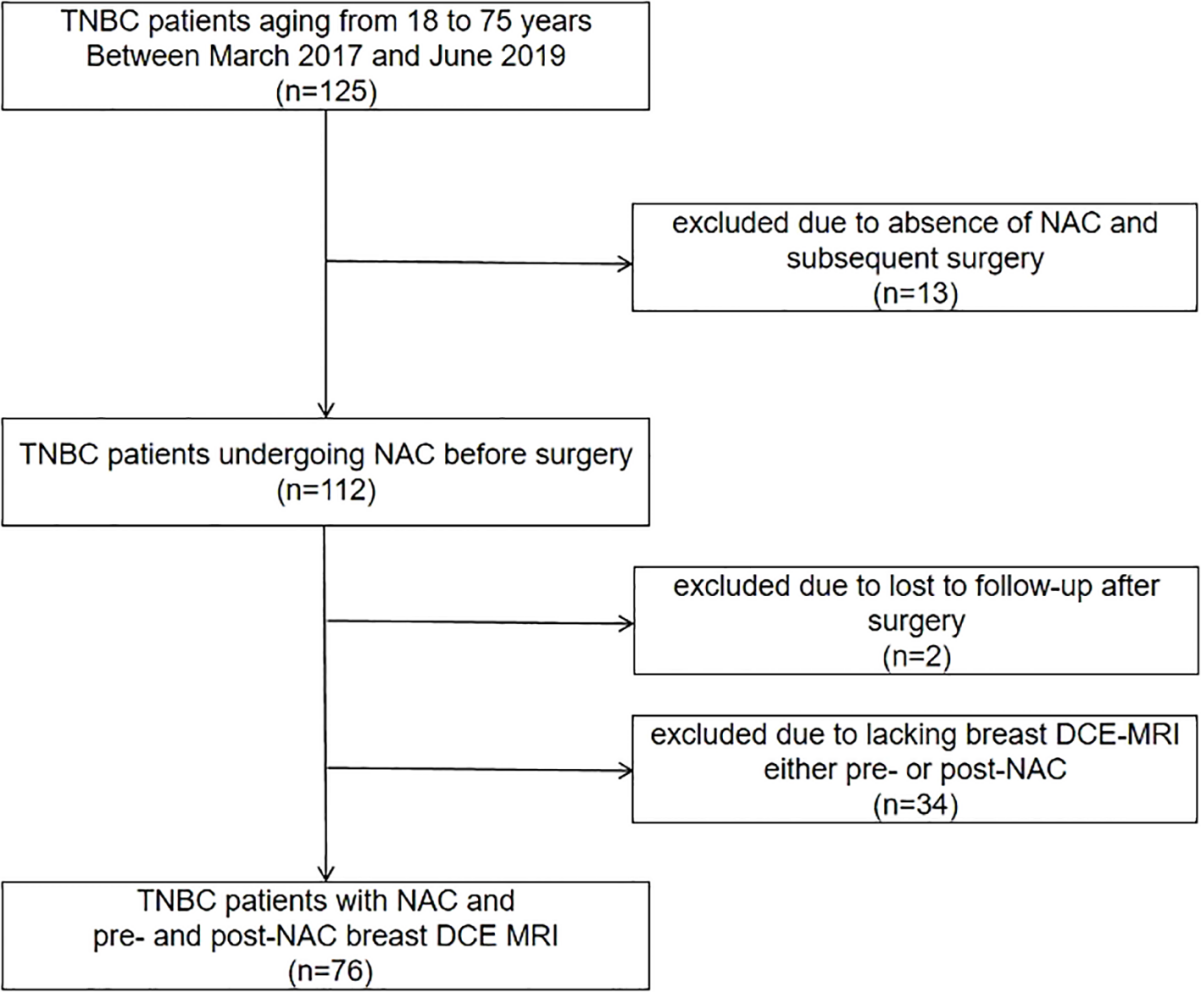
Flowchart for patient inclusion. TNBC, triple-negative breast cancer; NAC, neoadjuvant chemotherapy; DCE MRI, dynamic contrast-enhanced magnetic resonance imaging.

Between March 2017 and June 2019, 112 TNBC patients, 18 to 75 years of age, who received NAC before surgery, were candidates for this study. Thirty-four candidates were excluded due to a lack of MRI examination either before or after neoadjuvant therapy, and two candidates were excluded due to loss to follow-up after surgery (**Figure 1**). Finally, a total of 76 patients who met the criteria were included in the analysis, with 51.29 ± 9.53 years of age. Among them, 55 (72.4%) patients received dose-dense NAC. Finally, 46 (69.5%) patients achieved pCR and 30 (39.5%) patients did not. The median follow-up was 80 months (IQR: 64, 90) after surgery, and 13 (17.1%) patients developed recurrence. The patients’ characteristics are listed in **Table 1**.

**Table 1:** Characteristics of patients included in the study.

Characteristics	Categories	Mean	Std/percentage(%)/IQR
Age(years)		51.29	9.53
Menopausal status	yes	42	55.3
	no	34	44.7
Pathological type	IDC	66	86.8
	others	10	13.2
pre-NAC Tstage	T3-4	59	77.6
	T1-2	17	22.4
pre-NAC LN	pN+	28	36.8
	pN-	48	63.2
pre-NAC tumor size(cm)		2.5	(1.8,3.3)
NAC regimen	dose-dense	55	72.4
	conventional	21	27.6
Surgical methods	BCT	37	48.7
	Mastectomy	39	51.3
ypT stage	ypT0/Tis	31	40.8
	ypT+	45	59.2
ypN status	ypN+	15	53.6
	ypN-	13	46.4
post-NAC tumor size(cm)		1.1	(0.7,1.8)
pCR	yes	46	60.5
	no	30	39.5
Radiotherapy after surgery	yes	73	96.1
	no	3	3.9
Recurrence	yes	13	17.1
	no	63	82.9

std, standard deviation; IQR, interquartile range; IDC, invasive ductal carcinoma; others, invasive lobular carcinoma (ILC), mucinous carcinoma, apocrine carcinoma, and so on; NAC, neoadjuvant chemotherapy; BCT, breast-conserving surgery; pCR, pathological complete response.

### BPE on baseline breast MRI according to patient characteristics and MRI indexes

Excellent interobserver agreement was obtained for BPE evaluation with a weighted kappa coefficient of 0.904. The dichotomous BPE (minimal/mild vs. moderate/marked) on pre-NAC MRI and post-NAC MRI were both statistically associated with menopausal status (*P* = 0.045 and 0.015, respectively). The dichotomous BPE (minimal/mild vs. moderate/marked) on post-NAC MRI was also associated with ADC values, with minimal/mild BPE showing higher ADC values (*P* = 0.004). While BPE was dichotomized as minimal BPE and mild/moderate/marked BPE, those showing minimal BPE on post-NAC MRI had statistically less multifocality than those with mild/moderate/marked on post-NAC MRI, with *P*-values of 0.007 for multifocality on pre-NAC MRI and 0.011 for multifocality on post-NAC MRI. The BPE on pre-NAC MRI did not demonstrate a significant association with either clinicopathological or other MRI characteristics. The results are shown in **Tables 2**, **3**.

**Table 2:** Background parenchyma enhancement (BPE) on pre-NAC breast MRI according to patient characteristics and MRI indexes.

Patient characteristics	categories	Pre-NAC BPE				P	P'
		Minimal (n=40)	Mild (n=25)	Moderate (n=6)	Marked (n=5)		
Menopausal status	yes	26	13	3	0	*0.045*	0.072
	no	14	12	3	5		
Pathological type	IDC	34	22	6	4	0.556	0.438
	others	6	3	0	1		
pre-NAC Tstage	T3-4	29	21	5	4	0.534	0.258
	T1-2	11	4	1	1		
pre-NAC LN	pN+	16	13	1	1	0.091	0.883
	pN-	24	12	5	4		
NAC regimen	dose-dense	29	19	4	3	0.356	0.978
	conventional	11	6	2	2		
Surgical methods	BCT	19	16	2	2	0.341	0.483
	Mastectomy	21	9	4	3		
ypT stage	ypT0/Tis	15	22	2	2	0.509	0.642
	ypT+	25	13	4	3		
ypN status	ypN+	11	1	3	0	0.093	0.71
	ypN-	10	3	0	0		
pCR	yes	26	13	4	3	0.548	0.400
	no	14	12	2	2		
Radiotherapy after surgery	yes	39	23	6	5	0.621	0.46
	no	1	2	0	0		
Recurrence	yes	7	5	0	1	0.398	0.923
	no	33	20	6	4		
pre-NAC MRI indexes							
Maximum diameter(cm)		2.50(IQR:1.60,3.38)	2.60(IQR:2.30,4.65)	2.40(IQR:1.38,4.03)	2.30(IQR:2.05,2.50)	0.329	0.435
Morphology	mass	57	8	4	1	0.622	0.611
	non-mass	4	2	0	0		
Multifocality	yes	17	6	4	0	0.17	0.561
	no	44	4	0	1		
ADC		1.02(IQR:0.95,1.16)	1.08(IQR:0.94,1.14)	1.02(IQR:0.83,1.25)	0.94(IQR:0.86,1.02)	0.15	0.391
DCE curve type	inflow	6	1	0	0	0.249	0.229
	plateau	38	3	3	0		
	outflow	17	6	1	1		
post-NAC MRI indexes							
Maximum diameter(cm)		1.00(IQR:0.63,1.50)	1.50(IQR:0,80,2.40)	0.50(IQR:0,1.03)	0.80(IQR:0.35,1.95)	0.057	0.332
morphology	mass	48	6	2	0	0.528	0.169
	non-mass	5	1	0	0		
Multifocality	yes	15	6	3	0	0.077	0.420
	no	46	4	1	1		
ADC(10^-3^mm²/s)		1.38(IQR:1.06,1.62)	1.22(IQR:1.00,1.53)	1.64(IQR:1.28,1.99)	1.11(IQR:0.93,1.19)	0.434	0.255
DCE curve type	inflow	39	8	2	1	0.408	0.072
	plateau	15	1	1	0		
	outflow	1	1	1	0		

*P*: comparison between the dichotomous BPE group (minimal/mild vs. moderate/marked); *P′*: comparison between the dichotomous BPE group (minimal vs. mild/moderate/marked).

std, standard deviation; IQR, interquartile range; IDC, invasive ductal carcinoma; others, invasive lobular carcinoma (ILC), mucinous carcinoma, apocrine carcinoma, and so on; NAC, neoadjuvant chemotherapy; BCT, breast-conserving surgery; pCR, pathological complete response; ADC, apparent diffusion coefficient; DCE, dynamic contrast-enhanced.

**Table 3:** Background parenchyma enhancement (BPE) on post-NAC breast MRI according to patient characteristics and MRI indexes.

Patient characteristics	Categories	Post-NAC BPE				P	P'
		Minimal (n=61)	Mild (n=10)	Moderate (n=4)	Marked (n=1)		
Menopausal status	yes	36	6	0	0	* 0.015*	0.184
	no	25	4	4	1		
Pathological type	IDC	52	9	4	1	0.484	0.367
	others	9	1	0	0		
pre-NAC Tstage	T3-4	46	8	4	1	0.271	0.287
	T1-2	15	2	0	0		
pre-NAC LN	pN+	24	4	3	0	0.327	0.605
	pN-	37	6	1	1		
NAC regimen	dose-dense	44	8	3	0	0.424	0.601
	conventional	17	2	1	1		
Surgical methods	BCT	31	5	3	0	0.525	0.861
	Mastectomy	30	5	1	1		
ypT stage	ypT0/Tis	25	4	1	1	0.673	0.945
	ypT+	36	6	3	0		
ypN status	ypN+	11	1	3	0	0.073	0.668
	ypN-	10	3	0	0		
pCR	yes	37	6	3	0	0.661	0.963
	no	24	4	1	1		
Radiotherapy after surgery	yes	59	9	4	1	0.813	0.488
	no	2	1	0	0		
Recurrence	yes	8	2	3	0	0.398	0.075
	no	53	8	1	1		
pre-NAC MRI indexes							
Maximum diameter(cm)		2.50(IQR:1.750,3.25)	2.50(IQR:1.90,4.05)	2.65(IQR:2.35,4.15)	2.5	0.541	0.485
morphology	mass	57	8	4	1	0.655	0.338
	non-mass	4	2	0	0		
Multifocality	yes	17	6	4	0	0.051	*0.007*
	no	44	4	0	1		
ADC		1.02(IQR:0.94,1.16)	1.03(IQR:0.930,1.13)	0.99(IQR:0.83,1.10)	0.98	0.346	0.361
DCE curve type	inflow	6	1	0	0	0.596	0.186
	plateau	38	3	3	0		
	outflow	17	6	1	1		
post-NAC MRI indexes							
Maximum diameter(cm)		1.00(IQR:0.65,1.60)	1.25(IQR:0.65,2.95)	2.20(IQR:0.53,2.58)	0.7	0.475	0.176
morphology	mass	57	8	4	1	0.814	0.627
	non-mass	4	2	0	0		
Multifocality	yes	15	6	3	0	0.177	*0.011*
	no	46	4	1	1		
ADC(10^-3^mm²/s)		1.31(IQR:1.05,1.65)	1.29(IQR:1.099,1.49)	0.86(IQR:0.80,1.06)	1.07	*0.004*	0.120
DCE curve type	inflow	39	8	2	1	0.386	0.141
	plateau	15	1	1	0		
	outflow	1	1	1	0		

*P*: comparison between the dichotomous BPE group (minimal/mild vs. moderate/marked); *P*′: comparison between the dichotomous BPE group (minimal vs. mild/moderate/marked).

std, standard deviation; IQR, interquartile range; IDC, invasive ductal carcinoma; others, invasive lobular carcinoma (ILC), mucinous carcinoma, apocrine carcinoma, and so on; NAC, neoadjuvant chemotherapy; BCT, breast-conserving surgery; pCR, pathological complete response; ADC, apparent diffusion coefficient; DCE, dynamic contrast-enhanced.

**Figure 2:**
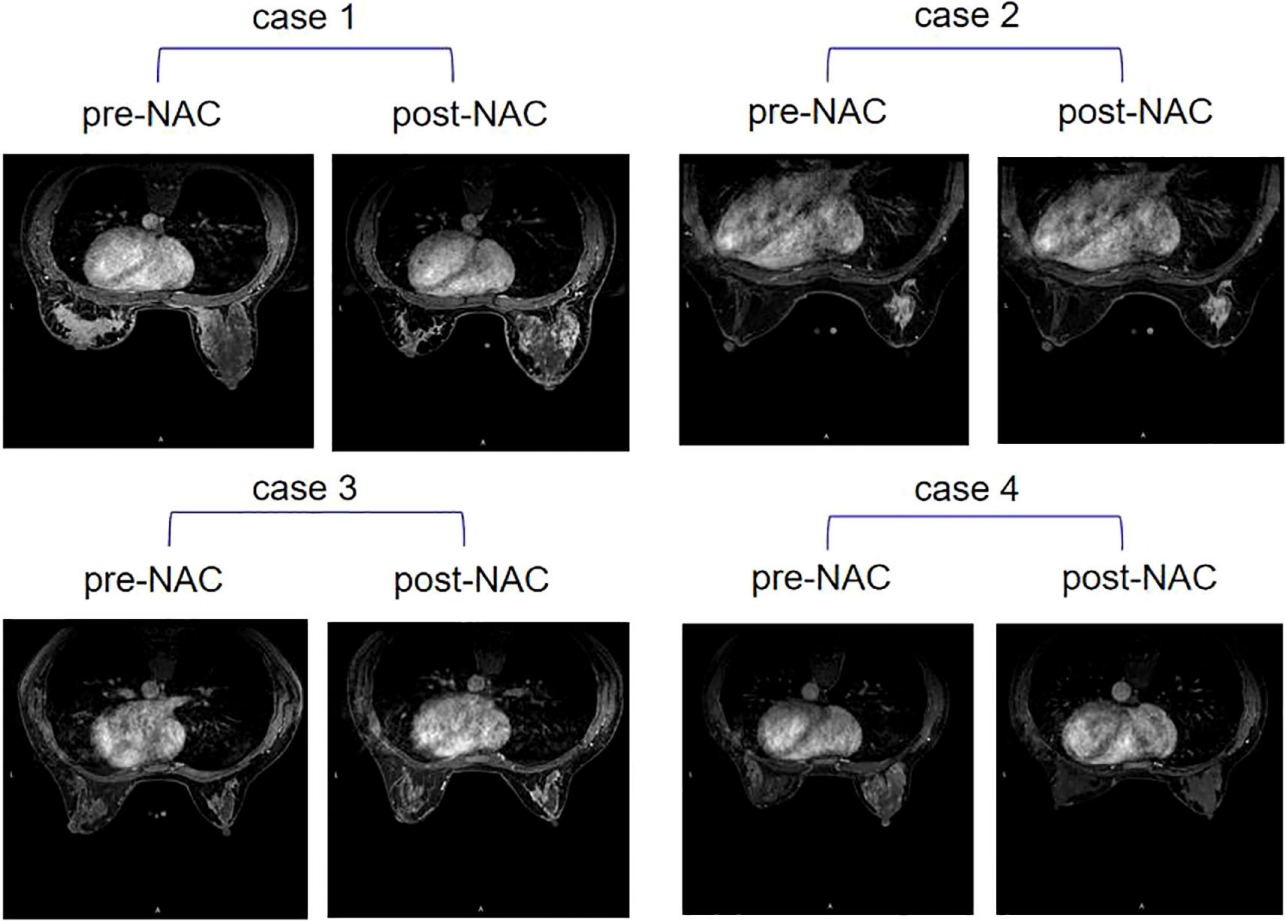
Four examples of contralateral BPE change after NAC. Case 1: BPE stayed high on pre-NAC MRI (moderate) and post-NAC MRI (moderate); case 2: BPE stayed low on pre-NAC MRI (minimum) and post-NAC MRI (minimum); case 3: BPE changed from low on pre-NAC MRI (mild) to high on post-NAC MRI (moderate); case 4: BPE changed from high on pre-NAC MRI (marked) to low on post-NAC MRI (minimum). BPE, background parenchymal enhancement; NAC, neoadjuvant chemotherapy.

The change of BPE after neoadjuvant therapy was analyzed according to pCR and recurrence (**Table 4**). The results showed that when a high BPE on pre-NAC MRI changed into low BPE on post-NAC MRI, no patient suffered recurrence (dichotomous BPE method 1), while if a low BPE on pre-NAC MRI changed into high BPE on post-NAC MRI, patients presented a high risk for recurrence (recurrence rate: 100%, dichotomous BPE method 1), and the *P*-value was 0.022. In dichotomous BPE method 2, similar results were observed (recurrence of BPE from high to low vs. BPE from low to high: 8.7% vs. 50%), but no statistically significant difference was observed (*P* = 0.23). However, BPE changes were not associated with pCR, neither in dichotomous BPE method 1 nor in dichotomous BPE method 2 (*P* = 0.406 and 0.611, respectively). Examples of BPE changes are shown in **Figure 2**.

**Table 4:** The change of BPE beween pre-NAC and post-NAC MRI according to pCR and recurrence.

Categories	dichotomous BPE method1			dichotomous BPE method2	
Post-NAC Pre-NAC	minimal/mild	moderate/marked	Post-NAC Pre-NAC	minimal	mild/moderate/marked
**minimal/mild**	63	2	**minimal**	38	2
**moderate/marked**	8	3	**mild/moderate/marked**	13	13
**pCR**	58.7(37/63)^a^	100(2/2)^b^	**pCR**	63.2(24/38)^a^	100(2/2)^b^
	75(6/8)^c^	33.3(1/3)^d^		56.5(13/23)^c^	46.2(6/13)^d^
**recurrence**	15.9(10/63)^a^	100(2/2)^b^	**recurrence**	16.7(8/48)^a^	50(1/2)^b^
	0(0/8)^c^	66.7(2/3)		8.7(2/23)^c^	30.8(4/13)^d^

NAC: neoadjuvant chemotherapy; pCR: pathological complete response; BPE: background parenchyma enhancement; **BPE method1:** Minimal/Mild vs Moderate/Marked; **BPE method2:** Minimal vs Mild/Moderate/Marked; ^a^: pCR or recurrence rate in both pre-NAC and post NAC low BPE; ^b^: pCR or recurrence rate in pre-NAC low BPE and post-NAC high BPE; ^c^: pCR or recurrence rate in pre-NAC high BPE and post-NAC low BPE; ^d^: pCR or recurrence rate in both pre-NAC and post NAC hign BPE.

### Survival analysis

**Figure 3:**
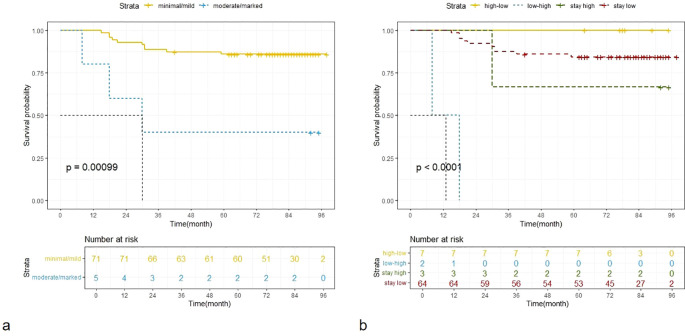
Kaplan–Meier curves for BPE on post-NAC MRI **(a)** and the change of BPE **(b)**. BPE, dichotomous background parenchymal enhancement; NAC, neoadjuvant chemotherapy.

Univariate Cox regression showed pre-NAC LN, surgical methods, ypT stage, ypN status, pCR, multifocality on pre-NAC and post-NAC MRI, DCE curve type, and BPE on post-NAC MRI and the change of BPE were significant factors for disease-free survival (**Table 5**). According to the multivariate Cox regression analysis, BPE and multifocality on post-NAC MRI, as well as pCR, were included in the final multivariate model, with adjusted HRs of 6.57, 3.65, and 7.27, respectively. Kaplan–Meier curves for BPE on post-NAC MRI and the change of BPE are shown in **Figure 3**.

**Table 5:** Univariate and multivariate Cox regression results according to recurrence.

Patient characteristics	Categories	univariate cox		multivariate cox	
		HR(95%CI)	P	HR(95%CI)	P
Menopausal status	no	1	0.313		
	yes	1.83(0.57,5.96)			
Pathological type	IDC	1	0.518		
	others	0.51(0.07,3.93)			
pre-NAC Tstage	T1-2	1	0.493		
	T3-4	1.70(0.38,7.64)			
pre-NAC LN	pN-	1	*0.004*		
	pN+	6.84(1.88,24.89)			
NAC regimen	dose-dense	1	0.123		
	conventional	2.38(0.79,7.14)			
Surgical methods	BCT	1	*0.020*		
	Mastectomy	5.94(1.32,26.84)			
ypT stage	ypT0/Tis	1	*0.040*		
	ypT+	3.24(1.05,9.96)			
ypN status	ypN-	1	*0.020*		
	ypN+	11.63(1.46,92.46)			
pCR	yes	1	0.036	1	*0.063*
	no	8.92(1.16,68.61)		7.27(0.90,58.77)	
Radiotherapy after surgery	yes	1	0.615		
	no	0.05(0,7091.30)			
pre-NAC MRI indexes					
Maximum diameter(cm)		1.01(0.97,1.05)	0.581		
Morphology	mass	1	0.961		
	non-mass	1.05(0.14,8.10)			
Multifocality	no	1	*0.010*		
	yes	4.70(1.45,15.30)			
ADC(10^-3^mm²/s)		0.36(0.02,6.65)	0.492		
DCE curve type	inflow	1	0.533		
	plateau	0.94(0.11,7.82)			
	outflow	1.77(0.21,14.72)			
pre-NAC BPE	minimal/ mild	1	0.461		
	moderate /marked	0.46(0.06,3.57)			
post-NAC MRI indexes					
Maximum diameter(cm)		1.03(0.99,1.07)	0.085		
morphology	mass	1	0.925		
	non-mass	0.91(0.12,7.02)			
Multifocality	no	1	*0.004*	1	*0.043*
	yes	5.78(1.78,18.80)		3.65(1.04,12.79)	
ADC(10-3mm²/s)		0.36(0.06,2.08)	0.255		
DCE curve type	inflow	1	*0.024*	1	
	plateau	0.66(0.14,3.08)	0.601		
	outflow	7.43(1.58,34.88)	0.011		
post-NAC BPE	minimal/ mild	1	*0.005*	1	*0.009*
	moderate /marked	6.56(1.79,24.03)		6.57(1.59,27.26)	
change of BPE	stay low	1	*0.001*		
	high to low	<0.001	0.982		
	low to high	39.90(6.48,245.58)	<0.001		
	stay high	2.24(0.29,17.51)	0.443		

HR: hazard ratio; IDC:invasive ductal carcinoma;others:invasive lobular carcinoma (ILC), mucinous carcinoma, apocrine carcinoma, and so on;NAC:neoadjuvant chemotherapy; BCT:breast-conserving surgery;pCR:pathological complete response; ADC:apparent diffusion coefficient; DCE:dynamic contrast-enhanced; BPE:background parenchymal enhancement.

## Discussion

This study provided evidence that moderate or marked BPE on post-NAC MRI indicated worse DFS, while BPE changing from high level (moderate/marked) to low level (minimal/mild) after NAC indicated better DFS. BPE on post-NAC MRI was an independent prognostic factor along with multifocality on post-NAC MRI and pCR status.

The physiological mechanisms of BPE are mainly related to angiogenesis, increased vascular permeability, and changes in the extracellular space (2). Additionally, fluctuations in hormone levels, particularly estrogen and progesterone, can significantly influence the physiological state of breast tissue in women. During the menstrual cycle, fluctuations in hormone levels will promote angiogenesis and increase vascular permeability within the breast tissue, resulting in obvious BPE on MRI (9). Therefore, breast MRI examinations should be conducted at the time that avoids the menstrual cycle. In this study, we also found that BPE was obviously higher in premenopausal patients. At the same time, extracellular matrix remodeling in the breast stroma and the release of inflammatory factors may also lead to an obvious BPE by enhancing local vascular permeability (14). Moreover, some studies have found that quantitative BPE varies between BRCA mutation carriers and non-carriers, as well as high-risk non-BRCA mutation carriers and non-high-risk non-BRCA mutation carriers (15, 16), which may be related to the abnormal proliferation of the breast stroma caused by DNA repair defects. However, there was one study that concluded a non-significant relation (17).

In recent years, studies have focused on the treatment response based on the BPE level. Many studies suggested a positive relationship between BPE level and residual tumor; obvious decreased BPE after neoadjuvant treatment could indicate higher rates of pCR, while higher BPE after neoadjuvant treatment usually means a poor response (18–20). Although BPE changes for pCR were reported in several articles, there is still controversy over it (21), as the study was carried out in hormone receptor (HR)-negative patients. Onishi et al. (22) found that non-suppressed BPE may be associated with inferior response to NAC in HR-positive patients, while in HR-negative patients, a similar tendency was observed without statistical significance. In our study, we did not detect a statistically significant association between BPE and pCR status (*P* = 0.548 for pre-NAC BPE and 0.661 for post-NAC BPE), which may be mainly due to the study population of TNBC patients.

From a physiological perspective, increased BPE may be associated with poorer prognosis, which has also been verified in some studies (5, 7, 23, 24). However, some studies have reached different conclusions, finding that contralateral BPE was not significantly associated with survival outcomes (25–27). These studies were usually conducted among patients with HR-positive breast cancer, or they included different molecular subtypes. There are a few related studies on triple-negative breast cancer. Consequently, the findings of this study offer valuable evidence regarding the potential of BPE as a prognostic indicator for TNBC. This research could potentially contribute to improve the prognostic evaluation and treatment of TNBC. If BPE can accurately identify patients with poor prognosis among TNBC patients, it may help manage targeted intensive treatment and timely adjustment of treatment plans. In addition, BPE could also serve as a means to monitor prognosis and recurrence for TNBC patients.

This study has some limitations. Firstly, the sample size is relatively small. The impact of the changes in BPE before and after neoadjuvant treatment on prognosis still needs to be verified in a large sample population. Secondly, BPE evaluation in this study relied on subjective judgment. Although subjective judgment is easy to operate and perfect interobserver agreement has been achieved, it should still be noted that there was a discrepancy in the evaluation of 6 cases out of 76 (7.9%). Thus, it is recommended to apply artificial intelligence-based quantitative measurement tools to eliminate the influence of the raters when used in clinical practice. Thirdly, this study was conducted among TNBC patients. In the future, it is necessary to investigate the impact of BPE changes on the prognosis among patients with hormone receptor-positive breast cancer. Moreover, by combining the physiological mechanisms of BPE, the heterogeneity of BPE changes can be more accurately understood in different molecular subtypes of breast cancer. Additionally, studies have shown that multiparametric MRI sequences can predict tumor proliferative activity and tumor immune microenvironment characteristics (28, 29). Therefore, research on the association between BPE and these biological markers will also help us understand the role of BPE in prognostic assessment.

In conclusion, this study suggested that BPE on post-NAC and its variation after neoadjuvant chemotherapy may be used to indicate the recurrence risk in TNBC patients.

## Data Availability

The raw data supporting the conclusions of this article will be made available by the authors, without undue reservation.
